# An Immunodominant Region of the Envelope Glycoprotein of Small Ruminant Lentiviruses May Function as Decoy Antigen

**DOI:** 10.3390/v10050231

**Published:** 2018-05-02

**Authors:** Marie-Luise Zahno, Giuseppe Bertoni

**Affiliations:** Institute of Virology and Immunology, Department of Infectious Diseases and Pathobiology, Vetsuisse Faculty, University of Bern and Federal Department of Home Affairs, CH-3012 Bern, Switzerland; marie-luise.zahno@vetsuisse.unibe.ch

**Keywords:** caprine arthritis encephalitis virus CAEV, small ruminant lentiviruses SRLV, decoy antigen, immunodominant epitope, escape, neutralizing antibody, lentivirus, original antigenic sin

## Abstract

(1) Background: Small ruminant lentiviruses (SRLV) persist in infected goats that mount a strong humoral immune response characterized by low neutralizing titers. In this study, we characterized the antibody response to SU5, a variable, immunodominant epitope of the envelope glycoprotein of SRLV. We tested the working hypothesis that the variability of SU5 reflects escape from neutralizing antibody. (2) Methods: Affinity purified anti-SU5 antibody were tested for their neutralizing activity to the homologous lentivirus. Virus culture supernatant—in native form or following sonication and filtration—was used to test the ability of free envelope glycoproteins to compete for binding in a SU5-peptide-ELISA. (3) Results: Anti-SU5 antibodies are not neutralizing, strongly suggesting that they do not bind intact viral particles. In contrast, shed envelope glycoproteins efficiently compete for binding in a SU5-ELISA, providing convincing evidence that the SU5 epitope is exposed only on shed envelope glycoproteins. (4) Conclusions: Our results show that the antibody engaging SU5 is not neutralizing and does not appear to bind to SU expressed at the surface of virus particles. We propose that SU5 is a potential decoy epitope exposed on shaded envelope glycoproteins, luring the humoral immune response in committing an original antigenic sin to a functionally irrelevant epitope.

## 1. Introduction

Caprine arthritis encephalitis virus (CAEV) and Maedi-Visna virus (MVV) are retroviruses belonging to the ovine-caprine lentivirus group of the genus lentivirus. These lentiviruses were long considered to be species specific pathogens of goats and sheep, respectively, but they were later shown to efficiently cross the species barriers and are now referred to as small ruminant lentiviruses (SRLV) [1,2]. SRLV do not induce overt immunodeficiency in the infected hosts and persist despite inducing a robust adaptive immune response, characterized by high antibody titers and a vigorous antiviral T cell immunity [3,4]. Especially in the case of the caprine arthritis encephalitis virus (CAEV), neutralizing antibody titers are low, and antibody is most likely implicated in SRLV induced pathological sequels such as arthritis, pneumonia, mastitis, and encephalitis [5].

The envelope glycoprotein (Env) is the principal target of neutralizing antibody, and its efficient masking by heavy glycosylation, characterized by the abundance of sialic acid, is considered to be the principal barrier blocking the binding of neutralizing antibody to SRLV particles [6].

Along with others, we mapped the linear B cell epitopes of the Env of CAEV [7,8]. SU5, one of the principal linear B cell epitopes detected in the surface portion of Env, is immunodominant and localized in a highly variable region [9,10]. We reasoned that the variability of this particular region could be the consequence of the immune selection applied by neutralizing antibody, as previously observed for an adjacent neutralizing epitope of MVV [11,12]. We tested this by analyzing the activity of affinity purified anti-SU5 antibody obtained from 3 goats infected 7 years before with the molecularly cloned virus CAEV-CO [13].

## 2. Materials and Methods

### 2.1. Animals

The three goats were selected from a group of six animals, previously infected with the CAEV-CO molecular clone [14]. These were the only 3 animals showing a consistent neutralizing activity, permitting us to perform the described experiments in controlled virus-serum pairs. Experiments performed under permission #57/95 and 23/97 (6 May 1997) obtained from the commission for animal experiments of the canton of Berne, Switzerland.

### 2.2. Synthetic Peptides

The following peptides were synthesized and purified by Primm, Milan, Italy. SU5-total: KVRAYTYGVIEMPENYAKTRIINRKK (env translation, position 7800–7877 [15]) SU5-variable: KEMPENYAKTRIINRKK (env translation, position 7830–7877 [15], the underlined Lysine (K) residue in this peptide was added to enhance binding to the ELISA plates). Affinity columns packed with the SU5-total peptide coupled with cyanogen bromide-activated Sepharose (2 mL) were purchased from Primm, Milan, Italy.

### 2.3. Antibody Affinity Purification

Antibody was purified as previously described [9]. Briefly, 10 mL of serum, obtained from each of the CAEV-CO experimentally infected goats, was mixed with 10 mL of binding buffer (ImmunoPure Gentle Binding Buffer; Pierce, Rockford, IL, USA), filtered through a 0.45-m-pore-size filter (Pierce, Rockford, IL, USA) and loaded onto the affinity columns (described in Section 2.2). The flow through was collected and the columns were washed with 30 mL of binding buffer before eluting the bound antibody with 10 mL of elution buffer (ImmunoPure Gentle Elution Buffer; Pierce, Rockford, IL, USA), collected in 1-mL fractions. According to the SU5-total ELISA results of the different portions, the following fractions were pooled and used: goat #01, fractions 2 to 8; goat #04, fractions 2 to 7; goat #13, fractions 2 to 4.

### 2.4. Peptide-ELISA and Anti-SU5 Titer

The SU5-total ELISA was performed, following a routine protocol as previously described [9]. A serial dilution of the affinity-purified antibody was prepared and tested to determine their anti-SU5 titers.

### 2.5. Antibody Avidity Measurements

The avidity index values of anti-SU5 antibody were measured as previously described; by testing the stability of antigen-antibody complexes following a wash step with 8 M urea [9].

### 2.6. Antibody Binding Inhibition Assays

To determine the binding specificity of the affinity-purified anti-SU5 antibody and to demonstrate the capacity of soluble SU molecules to compete for binding in a SU5-total peptide ELISA, competition experiments were performed as previously described [9]. Anti-SU5 antibodies were incubated with a molar excess of synthetic peptides (final concentration 5 μg per mL) or with cell culture supernatants of CAEV-CO infected GSM cells [16], before testing in a SU5-total ELISA. Additionally, to remove infectious virus and cell debris from the cell culture supernatants, these were sonicated (3 × 20 s, Sonifier 450 Branson, level 6, 50% pulsing on ice) and subsequently filtered (Vivaspin, 300,000 MW cutoff; Sartorius, Germany). As an additional control, anti-SU5 antibodies were incubated with CAEV gp135 (SU) at a final concentration 10 μg/mL. This glycoprotein was affinity-purified from culture medium of CAEV-infected GSM cells by the team of Dr. W.P. Cheevers, using a goat anti-SU monoclonal antibody (MAb) F7-299 [17].

### 2.7. Virus Neutralization

Neutralization assays were performed as described by McGuire and colleagues [18]. Neutralizing antibodies were detected by mixing 0.4 mL of heat inactivated serum (56 °C for 30 min), or affinity-purified anti-SU5 antibody, with 0.4 mL of MEM supplemented with 2% FCS and containing 10^3^ TCID50 of CAEV-CO. These mixtures were incubated for 1 h at 37 °C and 18 h at 4 °C. The non-neutralized virus was titrated on GSM cells in parallel with a virus sample incubated with a CAEV negative serum as control. This permitted us to calculate the virus neutralizing activity of the antibody preparations, expressed as % reduction of infectivity, compared to the negative control.

### 2.8. Sequence Alignments

Alignments were generated using Geneious version 10.2.3 (Biomatters Ltd., Auckland, New Zealand) [19].

## 3. Results

### 3.1. Antibody Titer and Avidity

The titer of the affinity-purified antibody was determined in a SU5-total ELISA as described previously [9]. Considering an arbitrary cutoff value of 0.3 optical density (OD), the titers were 3200, 1600 and 1600 for goat #01, #04 and #13, respectively. As expected, all anti-SU5 preparations were of high avidity. After washing the plates with 8 M urea, the avidity index values were 96%, 98% and 99% for goat #01, #04 and #13, respectively.

### 3.2. Binding Specificity of Anti-SU5 Antibody

**Figure 1:**
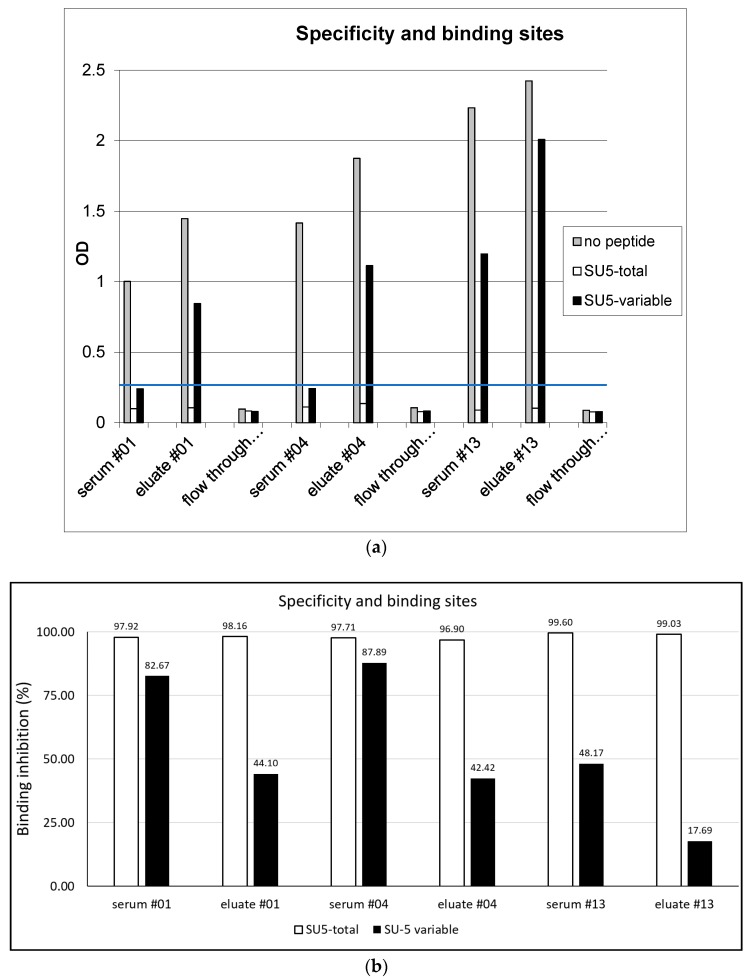
(**a**) Specificity and binding sites: Unfractionated sera, affinity purified anti-SU5 antibody present in the column eluate and antibody in the flow through were tested in a SU5-total peptide ELISA. The addition of SU5-total peptide completely suppressed binding, confirming the specificity of the reaction. A fraction of the antibodies present in the eluate bound to the variable part of SU5 and their binding was blocked by the addition of the SU5-variable peptide (confirmed in a SU5-variable ELISA). The remaining antibody in the eluate bound to a region overlapping the variable and constant regions. Anti-SU5 antibody is efficiently depleted from the column flow through. The blue line at OD 0.3 represent an arbitrary cutoff. (**b**) Specificity and binding sites: Pre-incubation of the antibody preparations with SU5-total peptide (open bars), or SU5-variable peptide (filled bars) inhibited antibody binding to SU5-total peptides coated on an ELISA plate (expressed as % binding inhibition, see (**a**)).

To define the specificity of both the reaction and the region of the SU5 peptide involved in antibody binding, we performed competition experiments with the SU5-total peptide and using a shorter version of the SU5 peptide encompassing only the highly variable region of SU5 (SU5-variable). As shown in Figure 1a, all 3 goats experienced a complete blockage in the binding of antibody to ELISA plates coated with the homologous peptide by the addition of a molar excess (final concentration 5 μg per mL) of the SU5-total peptide (inhibition >90%, Figure 1b). In contrast, the blockage was only partial by the addition of the SU5-variable peptide (Figure 1a,b). None of the anti-SU5 antibody preparations reacted to a consensus peptide encompassing the constant region of SU5, confirming the involvement of amino acid residues present in the variable region in antibody binding [9].

### 3.3. Neutralizing Activity

**Figure 2:**
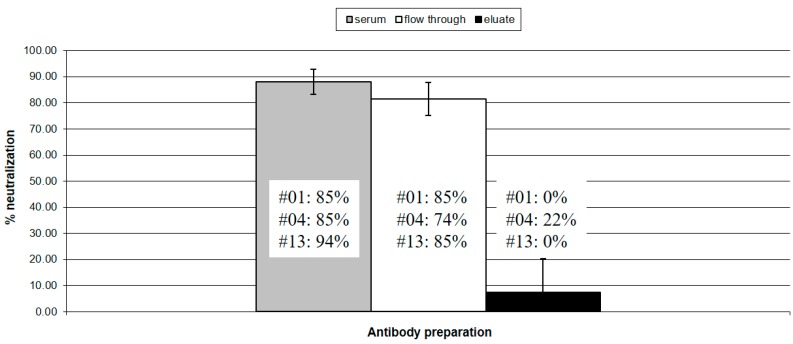
Virus neutralizing activity: The virus neutralizing activity of unfractionated sera (gray column), antibody present in the column flow through (white column) and affinity purified anti-SU5 antibody (black column) were tested in a standard virus neutralization assay using 10^3^ TCID50 of CAEV-CO. The histogram shows the neutralization activity of these antibodies, expressed as % neutralization of the input virus. An SRLV antibody negative serum was used as control. Standard deviations were calculated according to the results obtained with the three antibody preparations from goat #01, #04 and #13, respectively. Except for the eluate from goat #4, showing a borderline activity (22%), the affinity purified anti-SU5 antibody was completely devoid of neutralizing activity.

The three goats described in the previous paragraph were chosen because they showed a consistent neutralizing activity with titers between 8 and 16, typical of CAEV infected animals. We compared the neutralizing activity of the affinity purified anti-SU5 fraction with that of antibody contained in the unfractionated serum, the flow through of the affinity column and a negative control serum. As shown in Figure 1, the flow through was almost completely depleted of SU5 binding antibody, with remaining anti-SU5 ELISA titer of 50, 100, <50 for goat #01, #04 and #13, respectively. While the neutralizing activity of the flow through associated antibody was not significantly reduced in comparison to the unfractionated serum, the purified anti-SU5 antibody of goats #01 and #13 was devoid of neutralizing activity, with goat #02 showing a borderline neutralizing activity (Figure 2).

### 3.4. Binding of Anti-SU5 Antibody to Shed Envelope Glycoproteins

**Figure 3:**
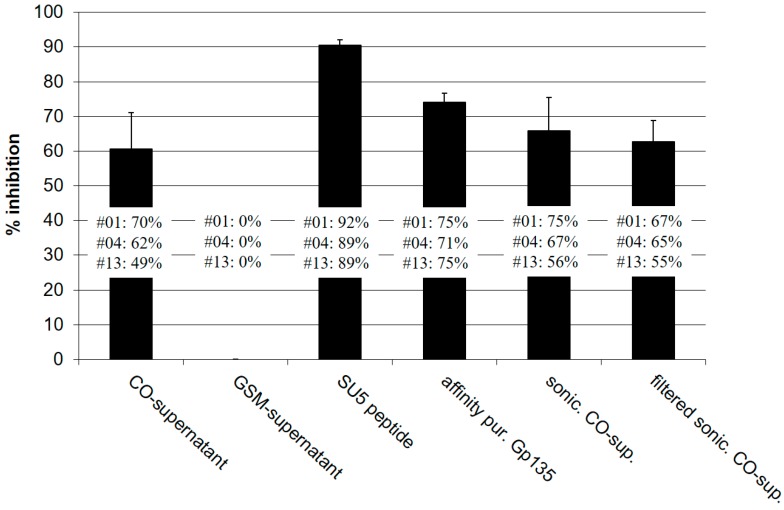
SU5 epitopes are available for binding in the supernatants of virus infected cells: Crude, sonicated and filtered supernatants of CAEV-CO infected GSM cells were tested in a SU5-total ELISA competition assay. Virus titers were 10^6.75^ and 10^3.25^ TCID50 for crude and sonicated supernatants, respectively. No infectious viruses were detected in the sonicated and filtered preparations. Supernatants of mock-infected GSM cells which did not show inhibitory activity compared to PBS were used as negative control. The same SU5 peptide used in ELISA was applied to compete for antibody binding (positive control). CAEV gp135 SU, purified by affinity chromatography, was added at a final concentration of 10 μg/mL. The affinity-purified antibody preparations obtained from goat #01, #04 and #13 were tested separately at 1:700 dilutions. The inhibitory activity of the different preparations (SU5 peptide, affinity purified Gp135, CO-supernatant, sonicated CO-supernatant and filtered-sonicated CO-supernatant) was calculated according to the values obtained with antibody preparations diluted in supernatant of mock-infected GSM cells (0% inhibition). Standard deviations were calculated according to the results obtained with the 3 antibody preparations. The binding of affinity purified antibody from goat #01 and #04 to SU5-total was more efficiently inhibited by the supernatants of CAEV-CO infected cells than the antibody from goat #13, confirming the differences in their binding characteristics described in the text and Figure 1 (inhibition >60% for #01 and #04 and <50% for #13).

In the absence of an evident reaction of anti-SU5 antibody with infectious CAEV particles, we argued that anti-SU5 antibody may react with virus debris present in the supernatant of virus infected cells, specifically in the form of soluble SU molecules. Indeed, we showed that the supernatant of CAEV-CO infected GSM cells contains molecules that interfere with the binding of affinity purified anti-SU5 antibody to homologous peptides in ELISA. This inhibitory activity was comparable to that of CAEV gp135 SU purified by affinity chromatography (a generous gift of Dr. W.P. Cheevers). The removal by filtration of infectious virus particles from these supernatants, previously sonicated to disrupt protein and virus clumps, did not affect the interfering activity, supporting the concept that the SU5 epitopes are exposed on virus debris and not viral particles (Figure 3).

### 3.5. Model of SU5 Availability to B Cells

**Figure 4:**
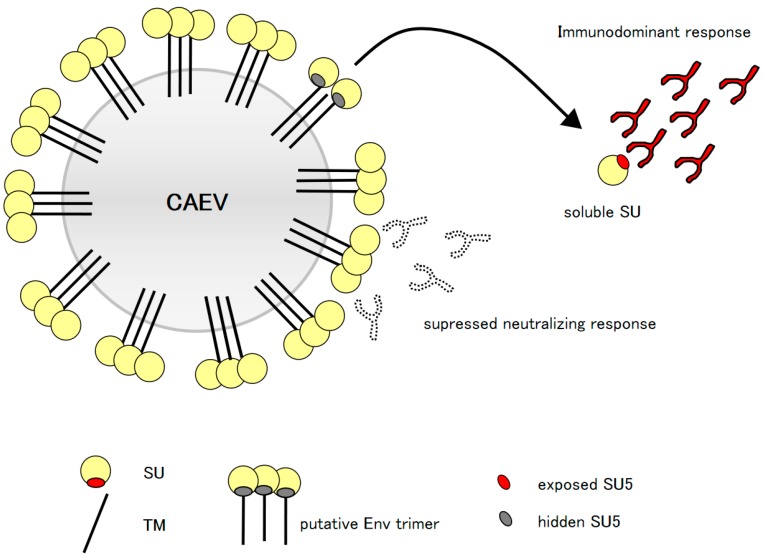
Model of the proposed “original antigenic sin”: Virus infected cells and virus particles release in the environment soluble forms of SU, unmasking the highly immunogenic SU5 region (red oval), which is normally hidden in the functional conformation of Env (gray oval). The released particles attract a strong, immunodominant, high avidity and polyclonal anti-SU5 antibody response that dominate the clonal hierarchy of the B cell immune response (antibody with black lines and red fill), potentially interfering with an efficient neutralizing response (antibody with dotted lines and white fill).

Based on these results we propose the following model (Figure 4). Upon infection, virus infected cells release SU, therefore exposing the highly immunogenic SU5 region. Independently of the infecting SRLV strains, which carry disparate amino acid sequences in the SU5 variable region, the humoral immune response to these peptides is strong, immunodominant and engages different B cell clones, as demonstrated by our competition experiments.

### 3.6. Analysis of SU-5 Sequences in a Particular Epidemiological Unit

The SU5-nucleotide and amino acid sequences of viruses isolated in a previously described epidemiological unit reflecting a trans-species infection between sheep and goats, showed that in this context, the SU5-sequences were surprisingly constant [20]. Indeed, the SU5 amino acid sequences obtained from two sheep (s7385 and s7631) and one goat (g6221) were unchanged at the amino acid level and showed only a silent nucleotide point mutation in a sequence obtained from sheep 7631 (Figure S1a). In contrast, the alignment of SU5 sequences derived from different epidemiological contexts showed the expected SU5 structure, with a constant frame region preceding a variable region (Figure S1b) [9]. Noteworthy, the amino acid sequence of the 1163M virus, belonging to the same phylogenetic subtype (SRLV-A4) as the abovementioned viruses but related to a different epidemiological context, showed several amino acid sequence mutations in its variable region (Figure S1b). Finally, the SU4 sequence of the virus isolated from sheep s7631, a region known to harbor a neutralizing epitope [21], showed several amino acid mutations (Figure S1c).

## 4. Discussion

Antibodies play a significant role in controlling lentivirus infections and in the HIV/SIV field, there is a consensus that broadly reacting, neutralizing antibodies are of pivotal importance in both controlling the persistent infection and in the future design of effective vaccines [22,23]. In this respect, CAEV infected goats are poor responders. They mount a robust humoral immune response against several virus proteins, particularly to Env, however this strong antibody response is poorly neutralizing and may contribute to immunopathogenic mechanisms of CAEV induced arthritis [5,16]. The abundant sialylation of Env was shown to mask important neutralizing epitopes that can be exposed to antibody neutralization by a neuraminidase treatment of virions [6]. Additional mechanisms diverting the B cell immune response from neutralizing epitopes may also be present. In HIV the B cell immune response tends to react to particular immunodominant regions of the Env molecules that permit an easy escape of the virus by mutation, conformational masking, or glycan shielding [22,23]. In the context of vaccine design, the masking of such epitopes with sugar residues was proposed as a strategy to direct the immune response to more relevant epitopes [24]. In this work we show that anti-SU5 antibody are abundant in persistently infected goats and can be efficiently purified by affinity chromatography. The eluted antibody was of high titer, high avidity and, as shown in Figure 1a,b, exhibited the same characteristic as the original sera—reacting to the variable region of the SU5 peptide, or a region encompassing the constant and variable region of SU5, but devoid of antibody reacting exclusively to its constant region. The performed neutralization experiments clearly contradicted our working hypothesis, showing that the purified anti-SU5 antibodies were completely devoid of neutralizing activity that remained associated with the flow through of the affinity column (Figure 2). Only the anti-SU5 antibody purified from goat #4 retained a residual neutralizing activity (22%) that may be attributed to contaminating neutralizing antibody. These data strongly suggest that anti-SU5 antibody is incapable of binding the functional form of Env expressed at the surface of infectious virus particles and infected cells. This is in accordance with a simple occupancy model of antibody neutralization suggesting that antibody binding with sufficient affinity to viral particles will have a neutralizing activity [25]. Moreover, the fact that the vast majority of CAEV infected goats mount a strong antibody response to SU5 but only a small minority consistently neutralize the infecting virus is strong indirect evidence supporting the lack of neutralizing activity in the anti-SU5 antibody fraction.

Confronted with these results, we reasoned that if anti-SU5 specific antibody does not bind infectious viral particles, it must be able to interact with soluble forms of Env. Competition experiments shown in Figure 3 demonstrate this point. Affinity purified gp 135, the surface subunits of Env (a generous gift of Dr. W. P. Cheevers), strongly inhibited the binding of affinity purified anti-SU5 antibody to the same peptide coated on ELISA plates [17]. This inhibition was comparable to the one achieved by adding an excess of free SU5 peptide to the eluted antibody. Noteworthy, the supernatant of CAEV-CO infected GSM cells also inhibited this binding. Sonication and filtration of this virus preparation eliminated the infectious virus without affecting the ability of the soluble proteins to compete for SU5 binding (Figure 3). The Env of SRLV is expressed as a precursor protein, subsequently cleaved by cellular proteases in its surface (SU) and transmembrane (TM) subunits that are non-covalently bound [26,27]. This interaction is weak, and SU is abundantly shed in the supernatant of infected cells, explaining the activity of our CAEV-CO infected GSM supernatant in competitive ELISA [28]. These results suggest that SU proteins shed in infected goats are most likely the antigen inducing the observed immunodominant humoral immune response to SU5. SU5 is positioned close to the carboxyterminal end of SU, directly adjacent to a β-strand structure part of the core inner domain of SU interacting with TM [29]. This strongly supports the concept that SU5 is not available for binding in the context of an intact SU-TM complex at the surface of viral particles and therefore cannot mediate a neutralizing activity. Based on this information we propose the model shown in Figure 4. SU5 becomes available to B cells only after SU is shed from viral particles or infected cells and induces a strong, albeit non-neutralizing humoral immune response. This promotes the expansion of B cell clones specific for SU5, potentially limiting those capable of producing neutralizing antibody, e.g., to a previously mapped neutralizing epitope adjacent to SU5 [21].

This suggests that SU5 may be a decoy epitope, luring the immune system to attack an irrelevant target, thereby neglecting important and less immunogenic neutralizing epitopes. Trujillo et al. provided strong evidence in support of this hypothesis by showing that the introduction of an artificial glycosylation site masking the SU5 epitopes increases the neutralizing titer of sera from immunized animals [30].

In previous experiments we observed that seronegative goats exposed to infected sheep carrying SRLV with known SU5 sequences showed different patterns of SU5 immune responses [10]. Goats born from certified CAEV free mothers seroconverted exclusively to the SU5 peptide corresponding to the infecting virus (SRLV-A4), while seronegative adult goats born to mothers previously infected with CAEV showed a strong anamnestic response to SU5 peptides of strains, such as SRLV-B1, different from the incoming virus (SRLV-A4) [10]. This is strong evidence supporting the concept that SU5 specific memory B cells are present in these otherwise seronegative goats. We interpret these results as manifestation of an “original antigenic sin” induced by the first encounter with an immunodominant SU5 epitope, as originally described for the influenza virus [31].

The induction of a strong humoral immune response to shed Env molecules and additional viral debris was postulated to be an important escape strategy used by HIV to divert B cells from more relevant neutralizing epitopes [32]. Recently, decoy epitopes were described for the porcine circovirus and the antibody response to such epitopes was shown to impair vaccine efficacy [33]. Replacing the decoy epitope with a protective epitope was shown to enhance vaccine efficacy [34]. This could not be applied to SU5, which is masked in the context of infectious viral particles.

Puzzling in this context is the high variability of the antibody binding portion of SU5 in different SRLV strains [7,8]. A simple explanation would be that this region is structurally tolerant to mutations that do not affect the interaction between SU and TM. We may also speculate that the immunodominant anti-SU5 immune response may favor virus replication. Suggestive evidence was found by sequencing 3 SRLV-A4 viruses isolated in a particular epidemiological compartment. The SU5 sequences of these viruses obtained from two sheep and one goat belonging to the same flock, but kept strictly separated, were surprisingly identical at the amino acid level, showing only a single synonymous mutation in this otherwise quite variable region (Figure S1a) [20]. This was not the case for the SU5 sequences obtained from different epidemiological contexts, showing several mutations in this region, even in a SRLV-A4 strain (goat isolate 1163M), phylogenetically very close to the viruses obtained from the abovementioned flock (Figure S1b). In contrast, the SU4 sequence of the virus isolated from sheep s7631, a region known to harbor a neutralizing epitope [21], presented several amino acid mutations (Figure S1c), suggesting the presence of a selective pressure exerted by neutralizing antibody that was obviously absent on the SU5 region. This freezing of the SU5 sequence in the context of an SRLV trans-species infection may suggest that the antibody response to SU5 confers an advantage to the viruses maintaining the original SU5 sequence. The fact that the SU5 portion of SU is not available for antibody binding excludes the possibility of an antibody-dependent enhancement of infection, parenthetically already tested and excluded by a previous study [35]. The immunodominance of SU5 may be related to the presence in its vicinity of immunodominant T cell epitopes favoring a B cell response and the marked affinity maturation observed [9,36]. The sequence conservation of SU5, observed in the abovementioned epidemiological compartment, may be an indirect consequence of the selective advantage of preserving such a putative T cell epitope. Indeed, a robust T helper cell response was previously shown to favor a higher viral load in vaccinated and challenged goats [37]. However, to prove this, a larger number of sequences should be analyzed, and the presence of the putative T cell epitope demonstrated.

## 5. Conclusions

A functional analysis of affinity-purified antibody directed to the immunodominant SU5 epitope of Env permitted us to definitively refute the working hypothesis linking the variability of this region to a potential escape from neutralizing antibody. We showed that this region is not available for binding in the context of intact viral particles but is readily exposed on soluble Env molecules. We propose that SU5 may be a decoy antigen inducing the immune system to commit an original antigenic sin to a functionally irrelevant epitope, potentially diverting the humoral response from the neutralizing epitopes of Env.

## Supplementary Materials

The following are available online at http://www.mdpi.com/1999-4915/10/5/231/s1, Figure S1a: Alignment of the SU5 region of viruses isolated from two sheep and one goat of the same farm, Figure S1b: Alignment of the SU5 region of different SRLV isolates, Figure S1c: Alignment of the SU4 region of goat g6221 and sheep s7385 and s7631.
