# Supplementary material for: An Immunodominant Region of the Envelope Glycoprotein of Small Ruminant Lentiviruses May Function as Decoy Antigen
Source: Viruses. 2018 May 2;10(5):231. doi: 10.3390/v10050231 (PMC5977224; doi:10.3390/v10050231)
Supplement: Supplementary file 1 [file viruses-10-00231-s001.pdf]

Supplementary Figure S1a-b-c:

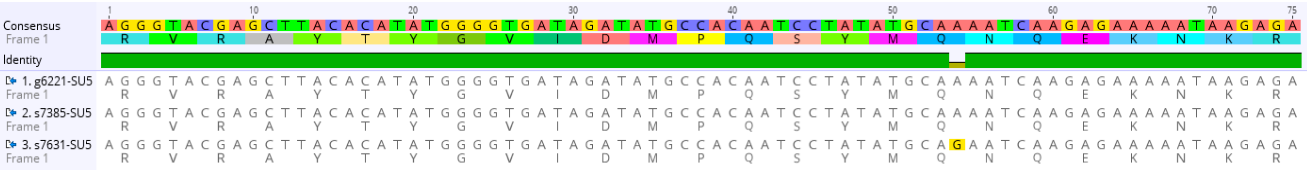

Figure S1a. *Alignment of the SU5 region of viruses isolated from two sheep and one goat of the same farm:* The sequence alignment shows a single synonymous mutation in the s7631-SU5 sequence (yellow G).

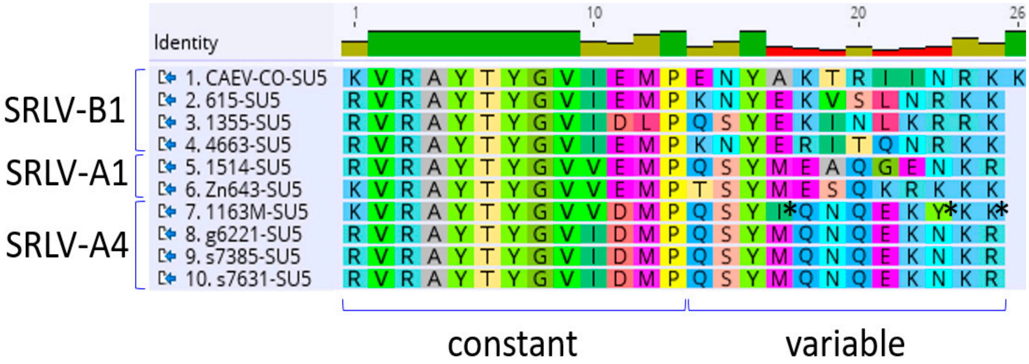

Figure S1b. *Alignment of the SU5 region of different SRLV isolates:* The SU5 amino acid sequences of the two prototypic SRLV sequences CAEV-CO (B1) and 1514 (A1) were aligned to different Swiss field isolate sequences. The constant and variable portions of SU5 are marked. Asterisk (*) point to the amino acid mutations in the 1163M SRLV-A4 isolate compared to the constant goat and sheep field isolates obtained from the same epidemiological unit (see Figure S1a).

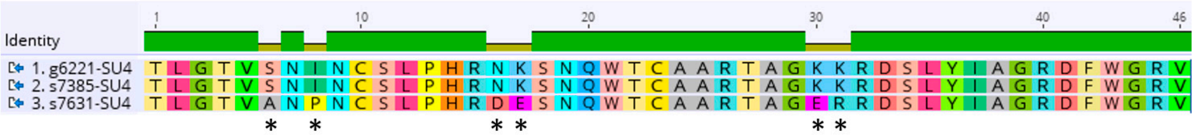

Figure S1c. *Alignment of the SU4 region of goat g6221 and sheep s7385 and s7631:* The SU4 amino acid sequences obtained from sheep s7631 shows 6 mutations (*) out of 46 amino acids, representing potential escape mutations from neutralizing antibody directed to this particular epitope [29]. In contrast, the variable portion of the SU5 region of these animals did not show mutations (see Figure S1a).
